# The Sterilization of Wounds by Physiological Agency

**Published:** 1918-09

**Authors:** 


### ABSTRACTS

### SURGERY

**The Sterilization of Wounds by Physiological Agency.** By Col. Sir A. M. Wright, Capt. A. Fleming, Capt. L. Colebrook. *The Lancet*, June 15, 1918.

A. From a study of the effects of serum deprived of its antitryptic power by addition of trypsin, serum rendered almost neutral by addition of acid, whole blood defibrinated on the growth of streptococcus, staphylococcus, B. coli, B. welchii, under aerobic and anaerobic conditions in capillary pipettes, the authors conclude:

1. Serum from normal blood and normal lymph constitutes for the vast majority of microbes met with in foul wounds a very unfavorable culture medium. Of the microbes encountered in wounds only the streptococcus, staphylococcus, and cultures of diphtheroid bacilli can grow in unaltered serum; and when we make a mineral implantation the streptococcus alone gives a growth. In other words the microbes of wounds fall into two categories: — serosaprophytes which grow in corrupted and serophytes in uncorrupted blood fluids. Therefore when a wound contains serosaprophytes there are corrupted discharges. If the wound can be flooded with wholesome serum which is kept uncorrupted, these serosaprophytes will disappear and the streptococci and staphylococci will persist.

2. Trypsinisized serum (present in all wound cavities and tissues when an exudate stands stagnant upon disintegrated leucocytes) produces an excellent medium for all bacteria.

3. Neutralized or partially neutralized serum (present in every condition of collapse) is a medium on which the ordinary, as well as the gas gangrene, bacteria grow readily. Gas gangrene microbes are serophytes of acidosed blood fluids.

4. Whole blood constitutes a medium of essentially the same qualities as the serum. In it, as a rule, only serophytes will grow, and usually only the streptococcus and staphylococcus.

B. From a study of the behavior of leucocytes in presence of bacteria the authors conclude that what is essential to the achievement of bacteriocidal effects is the employment of freshly emigrated leucocytes, the keeping of these alive and the removal of the excess of serum which would carry the bacteria out of reach. It is therefore obvious that the problem of sterilizing actual wound surfaces must be soluble along these lines.

C. On the possibility of sterilizing the superficies of a wound the authors believe there is no doubt, provided the surface has been washed quite clean from albuminous substances.

D. Practical Applications of Experiments: 1. Early primary suture stands on the same footing as primary suture following ordinary surgical operations. In both cases we are closing healthy tissues in which there is at most a minimal microbic implantation. In both cases, — except when we have dead spaces and allow these to fill with lymph and have in these a streptococcus implantation, — the body can very safely be trusted to deal with the few scattered microbes.

2. Treatment of foul wounds that have never been opened, cleansed, and resected, or wounds that have been closed without adequate cleansing, as well as originally clean wounds that have been neglected. The first step is to clean off the sloughs which favor every species of bacteria. Then when the surface is healthy get rid of the serosaprophytes. Triptic digestion accomplishes the first — whereas the second is accomplished by impouring of wholesome lymph. This digestion can be accelerated by soaking the wound with 5 o/o salt which also acts as a lymphagogue and stops the serosaprophytic infection. The serophytic infection is held in check so long as the concentration of salt in the discharges is maintained at a high level.

3. Treatment of gas gangrene infections. Necrotic tissue here should be removed with knife; altered lymph should be replaced with wholesome lymph. After excision, therefore, a local lymphagogue (hypertonic salt solution) is clearly indicated. Antiseptics are of no value whatsoever, as they can not penetrate sloughs or pockets of pus or infected tissues, nor do they influence that corruption of the discharges which favor the growth of every species of microbe.

4. Treatment of clean wounds. Here the sterilization must be completed by destruction of serophytic bacteria. Recourse may be had to the patient's leucocytes or to chemical antiseptics.

E. The authors conclusions are as follows:

1. It has been erroneously inculcated that every wound should be sterilized before closure; and that, therefore, primary suture should be avoided and secondary suture undertaken only after a course of antiseptics. There is now no question, with respect to primary suture, that the wound taken after early surgical cleansing and resection is as good as sterile; and, with regard to secondary suture, undertaken with a wound in good condition and a purely serophytic infection, that such operative procedure, provided it leaves behind no infected dead spaces, directly contributes to sterilization.

2. It has been taught that we should judge of the fitness of the wound for closure by necro-pyo-cultures and direct microscopic examination of the pus. We have learned that it would be infinitely more reasonable to base our judgments upon the results of bio-pyo-culture.

3. It has been taught that suture cannot be successful in a wound containing a hemolytic *Streptococcus pyogenes*. We have seen that leucocytes can, given proper conditions, successfully combat this, and of course all other streptococci; and that these conditions can be realized in connection with the suture of wounds.

4. It has been taught that for the removal of sloughs from foul wounds chemical solvents are required. We have learned that sloughs can be removed by tryptic ferment set free from disintegrated leucocytes, and that the liberation of this ferment can be greatly accelerated by breaking down the leucocytes in the discharges with hypertonic saline solution.

5. Lastly, it has been taught in connection with antiseptics that sterilization is obtainable only by continuous or very frequently repeated application. We have learned that there is nothing to prevent any part of a wound surface, which has been washed quite clear of albuminous matter, from being sterilized by a single application of antiseptics.

